# The effect of feedback to general practitioners on quality of care for people with type 2 diabetes. A systematic review of the literature

**DOI:** 10.1186/1471-2296-10-30

**Published:** 2009-05-06

**Authors:** Trine Lignell Guldberg, Torsten Lauritzen, Jette Kolding Kristensen, Peter Vedsted

**Affiliations:** 1 Department of General Practice, Institute of Public Health, Aarhus University, Bartholins Alle 2, 8000 Aarhus C, Denmark; 2 Research Unit for General Practice, Institute of Public Health, Aarhus University, Bartholins Alle 2, 8000 Aarhus C, Denmark

## Abstract

**Background:**

There have been numerous efforts to improve and assure the quality of treatment and follow-up of people with Type 2 diabetes (PT2D) in general practice. Facilitated by the increasing usability and validity of guidelines, indicators and databases, feedback on diabetes care is a promising tool in this aspect. Our goal was to assess the effect of feedback to general practitioners (GPs) on the quality of care for PT2D based on the available literature.

**Methods:**

Systematic review searches were conducted using October 2008 updates of Medline (Pubmed), Cochrane library and Embase databases. Additional searches in reference lists and related articles were conducted. Papers were included if published in English, performed as randomized controlled trials, studying diabetes, having general practice as setting and using feedback to GPs on diabetes care. The papers were assessed according to predefined criteria.

**Results:**

Ten studies complied with the inclusion criteria. Feedback improved the care for PT2D, particularly process outcomes such as foot exams, eye exams and Hba1c measurements. Clinical outcomes like lowering of blood pressure, Hba1c and cholesterol levels were seen in few studies. Many process and outcome measures did not improve, while none deteriorated. Meta analysis was unfeasible due to heterogeneity of the studies included. Two studies used electronic feedback.

**Conclusion:**

Based on this review, feedback seems a promising tool for quality improvement in diabetes care, but more research is needed, especially of electronic feedback.

## Background

In our efforts to improve and assure the quality of care in general practice, information technology is becoming increasingly used [1]. Quality improvement tools for diabetes, often delivered electronically, include general information and clinical guidelines, feedback on the quality of care as well as patient information letters. Furthermore, many general practitioners (GPs) use electronic patient's records. The use of information technology in general practice is facilitated by the increasing numbers and validity of clinical databases [2]. These databases make it possible to extract accurate information in an easy and low cost way.

In 2006, a Cochrane review concluded that "Audit and feedback can be effective in improving professional practice (...) the relative effectiveness of audit and feedback is likely to be greater when baseline adherence to recommended practice is low (...)[3].

Recent published figures [4] show that 88% of native Danish PT2D had their Hba1c measured at least once during year 2003; for eye examinations the figure was 33% increasing to only 61% over a four year period. 70% of the native Danish PT2Ds had an annual serum cholesterol measurement. These figures show that there is room for improvement when it comes to caring for PT2Ds.

Recent reviewing of prompting clinicians about preventive care measures have revealed a modest consequence, with cardiac care and smoking cessation reminders being most effective [5]. The aim of this paper was to review the available literature on the effect of feedback on diabetes care in general practice, and more specifically whether there was an effect of feedback to GPs on process and outcome measures for the quality of care for PT2D and to what extent electronic feedback had been investigated. We define feedback as "any summary of clinical performance of health care over a specified period of time". Electronic feedback is defined in the same way but delivered to the end user via computer [3].

## Methods

A search was conducted using October 2008 updates of Medline (Pubmed), Cochrane library and Embase databases. Only English written papers were included in this review. The searches were not time period limited

Separate searches were conducted on the following MeSH and in-text terms:

#1: Type 2 Diabetes, #2: General Practice, #3: Family medicine, #4: Family medicin, #5: Family Practice, #6: Feedback, #7 Decision support, #8: Reminder system.

These searches were then combined into: #9: (#2 or #3 or #4 or #5) and #10: (#6 or #7 or #8). Finally, all searches were combined into #11: (#1 and #9 and #10).

The search and assessment strategies used in this review were based on course material from the DIRAC course of Systematic reviews and Meta Analysis held in Copenhagen in August 2007 [6]. However, as the aim was not to perform a meta-analysis and a thorough rating of the evidence, only one researcher did the searching and primary assessments. First author (TLG) did all the searches and assessing and discussed any doubts with the rest of the research team. Further, all results were scrutinised by the research group.

The search term "Type 2 Diabetes" was chosen above "Diabetes Mellitus" to fulfil the aim of this review, which does not consider "Type 1 Diabetes". Even though the Mesh term "Type 2 Diabetes" is relatively new, it realised papers published between 1994 and 2005. One unpublished study was considered for inclusion, based on published work concerning the methodology of the study. However, correspondence with the research team running the trial was inconclusive and left the study irrelevant for reviewing. Reviews that appeared in the searches were used in order to check the reference lists for includable randomized trials. The term "reminder system" was included as it was discovered that this term often covered what we defined as feedback. To distinguish between reminder system and feedback we assessed each of the retrieved papers separately.

The reference lists of the papers retrieved were checked for includable papers as were the "related articles" facility in PubMed. An initial database search retrieved six papers and a search of the related articles and reference list included an additional eight papers. Ten of the14 papers complied with the following inclusion criteria:

• Randomised trial

• Study concerning diabetes

• Study set in general practice

• Interventions aiming at using feedback to GPs on diabetes care.

The remaining four articles were excluded because they did not concern feedback.

### Assessment

The included studies were assessed according to predefined criteria:

• Aim of the study (How well did design cohere with study aim)

• Method of evaluation (How well did methods of analysis cohere with study design?)

• Format of feedback/intervention (Electronic or not)

• Effect measure divided into:

◦ Process measures, i.e. are things done? Is, e.g. HbA1c measured according to the study-based guidelines?

◦ Outcome measures, i.e. has the level improved? Have, e.g. the HbA1c levels improved according to the aims of the study-based guidelines?

• Effect of feedback

• Data collection

• Problems identified in randomization, sampling, blinding and drop-out

## Results

The ten papers presented ten different studies. Of these, six were conducted in USA, one in New Zealand, one in The Netherlands and two in Scandinavia. The papers were published between 1994 and 2005, of these two before 2000. The duration of the trials varied between two months and six years, with a median duration of 12 months.

Table 1 summarizes the studies on design, number of participants, trial duration and data collection. The method of evaluation and effect measures are summarized in Table 2 and statistical significant effect measures are summarized in Table 3.

**Table 1:** Summary of intervention design, trial duration

**Author/Country**	**Intervention design**	**Trial duration**	**No. patients (intervention/control)**	**No. doctors (intervention/control)**	**Data collection**
Phillips [7]/USA. 2005	Clinicians were randomized to be controls or receive either computerized reminders, feedback on performance from specialized endocrinologist or both interventions. Feedback sessions with endocrinologist focused on individual provider actions or outcomes of specific patients.	3 years.	4138 (3155 divided into 3 groups/983)	345 (?/?)	Research assistants encountered with all the patients to collect data.
Sequist [14]/USA. 2005	Clinics were randomized so that physicians received either evidence-based electronic reminders within their patients' electronic medical record or usual care. There were five reminders for diabetes care.	6 months	4549 (2924/3319). No drop out.	194 (92/102)	Data collected using existing databases.
Frijling [8]/NL. 2002	The intervention group received feedback reports and support from a facilitator; the control group received no special attention.	23 months	2859 encounters (?/?) The exact number of patients is not reported.	124(62/62) Drop out: 2,4%	GPs filled out encounter forms and questionnaires about patient characteristics.
Lobach [9]/USA. 1994	Clinicians were randomized to receive either a special encounter form with the computer-generated guideline recommendations or a standard encounter form.	6 months	359 (?/?).	58 (?/?).30 doctors included in analysis.	Researcher collected data by chart review.
Nilasena [10]/USA. 1995	Internal medicine residents were randomised to receive either computer-generated patient-specific reminders about the diabetes guidelines or a nonspecific report.	6 months	480 (?/?). 164 included in analysis (excluded: 66%)	35 (?/?)	Researcher collected data by chart review.
Hetlevik [15]/N. 2000	Clinics were randomized to receive either electronic clinical reminders within the electronic patient records and reports on diabetes care in general or no special attention.	18 months	1034 (499/535)	53 (24/29)	Data was collected in GPs records. A questionnaire was distributed among the participating GPs
Kenealy [11]/NZ. 2005	Four intervention arms: patient reminders, computer reminders, both reminders, and usual care. The patient reminder was a diabetes risk self-assessment sheet filled in by patients and given to the doctor during the consultation. The computer reminder was an icon that flashed only for patients considered eligible for diabetes screening. Clinics were units of randomization.	2 months	5628. (4756 divided in 3 groups)/872)	112 (83/29). Drop out 13,4%.	GPs answered on encounter forms whether or not they had screened for diabetes.
Kiefe [16]/USA. 2001	Physicians were assigned to either a multimodal improvement intervention, including chart review and physician-specific feedback or an identical intervention plus achievable benchmark feedback.	3 years	2978 (?/?)	70 (35/35)	Data obtained from chart review by researchers
de Fine Olivarius[12]/DK. 2001	Clinicians were randomized to either controls or structured care comprising of regular follow up and goal setting of specific patients. This was supported by prompting of doctors, clinical guidelines, feedback and continuing medical education.	6 years	944 (459/415)	484 (?/?). Drop out: 40,5% in both control and intervention group.	Data collected through GPs, through eye doctors, via laboratory databases and via questionnaires to patients.
Glasgow [13]/USA. 2004	Physicians were randomised to receive either a CD ROM-assisted diabetes care enhancement program were patients were invited to complete the computerized Diabetes Priority Program touch screen assessment and feedback procedure, or to receive no special attention.	6 months	886 (469/417)	52 (24/28)	Data primarily collected via patients. Some data collected in laboratory database.

? = Information unavailable in paper

**Table 2:** Evaluation methods and effect measures used in the ten studies

	**Evaluation method**		**Effect measures**	
	
**Study**			**Proces**	**Outcome**
Phillips [7]	Pre- and post intervention mean group differences on patients in intervention vs. control groups.			†Mean Hba1c. Mean Ldl Cholesterol. Blood pressure.
Sequist [14]	Both summary and individual composite endpoints		†Cholesterol measured, Hba1c measured. Eye exam. ACE inh if hypertension.	†Odds of receiving recommended diabetes care *
	Summary endpoint: diabetes reminders resulting in action/diabetes reminders in total Individual endpoints: No. of appropriate diabetes health services received by pt/total no. of diabetes health services pt			
Frijling [8]	Mean compliance rate for each performance indicator at baseline and the mean change from baseline.		†Foot exam. †Eye exam. BMI. Medication. Measured blood press. Anti diabetic treatment. Scheduled next appointment	
Lobach [9]	Mean compliance rate for each performance indicator at baseline and the mean change from baseline. Composite outcome of all performance indicators at baseline and the mean change from baseline.		†Physical exam †Urine protein measured. †Cholesterol measured. Eye exam. Influenza vac. Pneumococ vac. Foot exam. Hba1c measured.	†Median level of compliance * †Median adherence rate of the clinicians *
Nilasena [10]	Mean compliance rate for each performance indicator at baseline and the mean change from baseline.		Foot exam., physical exam. Hba1c measured. Urine protein measured. Cholesterol measured. Eye exam. Influenza vac. Pneumococ vac.	
Hetlevik [15]	Mean group differences in fractions of patients without registrations (process evaluation).	Mean group differences in variables (patient outcome evaluation)	Registered smoking habits. BMI. Blood pressure measured. Hba1c measured. Cholesterol measured.	†Blood pressure. Number of smokers. BMI. Mean Hba1c. Mean cholesterol.
Kenealy [11]	Was/not tested for blood glucose if eligible.		†Blood glucose measured	
Kiefe [16]	Proportional changes in the receiving of guideline specific diabetic services.		†Influenza vac. †Foot exam. †Hba1c measured, cholesterol measured. Triglycerides measured	
de Fine Olivarius [12]	Pre- and post intervention mean group differences on patients in intervention vs. control groups.		Measurement of blood glucose- Measurement of Hba1c. Measurement of blood pressure. Measurement of cholesterol. Measurement of triglycerides	†Level of plasma glucose. †Level of Hba1c. †Level of blood pressure. †Cholesterol Mortality. Diabetic retinopathy. AMI. Stroke. Angina pectoris. Claudicatio, Amputation.
Glasgow [13]	Effect of system evaluated on 2 primary outcomes: number of recommended laboratory screenings and recommended patient-centred care activities completed. Secondary outcomes were evaluated using the Problem Areas in Diabetes scale and the Patient Health Questionnaire.		†Foot exams. †Nutrition counselling Blood pressure measured. Eye exam. Foot exam. Micro albumin.	†No of recommended lab assays * †Patient-centred aspects of diabetes care received *

† = Significant positive changes.

* Composite outcome

**Table 3:** Effect measures in trials with significant changes

**Measure**	**Author**	**Effect**
**Proces measures (pm) (no. of trials in which pm was included)**		

Foot examination	Kiefe [16]	OR 1,33; 95%CI 1,05–1,69
	Glasgow [13]	RR 4,38; 95%CI 2,42–7,91; boc
	Frijling [8]	OR 1,68; 95%CI 1,19–2,39
Hba1c measurement	Kiefe [16]	OR 1,33; 95%CI 1,04–1,69
Eye examination	Frijling [8]	OR 1,52; 95%CI 1,07–2,16.
	Glasgow [13]	RR 1,79; 95%CI 1,20–2,68; boc
Influenza vaccination	Kiefe [16]	OR 1,57; 95%CI 1,26–1,96
Cholesterol measurement	Lobach [9]	duc, significant positive changes reported in text
	Sequist*[14]	HR 1,41; 95%CI 1,15–1,72
Blood glucose measurement	Kenealy [11]	Pt reminders OR 1,72; 95%CI 1,21–2,43
	Kenealy [11]	Computer reminders OR 2,55; 95%CI 1,68–3,88
	Kenealy [11]	Both reminders OR 1,69; 95%CI 1,11–2,59
Dietary advice	Glasgow [13]	p < 0,001;duc
Micro albumin measurement	Lobach [9]	duc, significant positive changes reported in text
	Glasgow [13]	RR 3,97; 95%CI 2,22–7,10; boc
**Outcome measures (OM) (No. of trials in which OM was included)**		
Level of blood pressure	De fine Olivarius [12]	Δ-5 mmHg; 95%CI -7,6 to -2,4
	Hetlevik* [15]	Δ-2,3 mmHg; 95%CI -3,8 to -0,8
	Phillips [7]	OR 1,19; 95%CI 1,07–1,32
Level of Hba1c.	Phillips [7]	OR 1,18; 95%CI 1,03–1,34
	De fine Olivarius [12]	Δ-0,056%; 95%CI -0,081% to -0,031%
Level of cholesterol.	De fine Olivarius [12]	Δ-0,15 mmol/l; 95%CI -0,29 to -0,02
**Aggregated measures (AM) (No. of trials in which AM was included)**		
Odds of receiving recommended care	Sequist*[14]	OR 1,30; 95%CI 1,01–1,67
Compliance rate	Lobach [9]	32% vs. 15,6% (p = 0,02*), duc
Number of recommended lab assays	Glasgow [13]	F = 9,90, p < 0,001; duc
Patient Sataibisfaction	Glasgow [13]	F = 25,2, p < 0,001;duc

boc = Based on own calculations on data extracted from paper. duc = Data unavailable for further calculations. OR = Odds ratio. HR = Hazard ratio. RR = Relative risk. * = Electronic feedback

### Aim of the studies

All studies aimed at improving the GPs' adherence to the diabetes guidelines in order to improve the care for PT2D. All the studies leaned on existing guidelines or developed guidelines as a part of the intervention.

### Format of feedback (interventions)

Nine studies used feedback distributed to the GPs in printed format [7-14] and two used electronic feedback [14,15]. One study using electronic feedback also distributed the reports on paper [12]. Eight studies generated patient specific feedback [7,9-12,14,15] while two generated aggregated feedback for specific practices or physicians [8,16].

Study design used to evaluate feedback varied according to whether the feedback was the single aim of evaluation, or the feedback was only part of a larger intervention with the aim of evaluating other means of quality improvement as well. Six study groups concentrated only on feedback in their interventions [9,10,13-16]. Of these, one added benchmarks to the feedback in one intervention arm [16], and one let the patients fill in the feedback reports in the GP's waiting room [13].

Three studies compared regular feedback to other interventions, or combined feedback with other means of support: One combined feedback with outreach visits [8]; one compared feedback with face to face evaluation with an endocrinologist [7]. One study compared feedback filled in by patients in the waiting room and delivered in paper format with a computer reminder consisting of a blinking icon on the GP's computer screen [11].

One study categorised the intervention as multifaceted, and feedback to the GPs was part of the intervention [12].

### Effect measures

All studies used process measures that were part of routine diabetes management, i.e. measuring blood glucose and serum cholesterol. Some used composite outcomes such as compliance rates, measuring the compliance of the GPs to the diabetes guidelines, while others focused on specific process or outcome measures (Table 2).

One study differed from the others by using groups of endpoints defined as: PT2D satisfaction (PS), Diabetes specific quality of life (Dsql) and Depression severity symptoms (Dss) evaluated by using validated questionnaires [13]. These endpoints were based on questionnaires and process measures combined.

### Data collection

Data were collected via chart review [9,10,15,16], encounter forms filled in by the participating GPs [8,11], databases [14], or by combining the methods [10,11]. In one study, all included patients were examined by one data collecting member of the research team [7].

### Effect of feedback

One study showed no statistical, significant changes [10]. Nine papers reported a total of 23 statistically significant positive changes [7-9,11-16]. Fifty one variables in nine papers did not change statistically significantly. No negative changes were reported (See Table 3). The process measure most often improved was foot examination. Aggregated outcomes improved significantly in three trials [9,13,14].

### Long term effects

Our searches revealed 3 interventions which lasted more than three years [7,12,16]. All three interventions showed significant positive results. In one trial, the researchers concentrated on process measures. They showed significant results on foot examination, Hba1c measurement and influenza vaccination [16]. The two other trials both included effect measures, and both showed significant improvements on Hba1c levels, serum cholesterol levels and blood pressure levels [7,12]. One long term trial included effect measures such as mortality, diabetic retinopathy, AMI, stroke, angina pectoris, claudicatio and amputation, but did not manage to show significant changes on any of these measures over a period of six years.

### Methodological problems identified

In two studies, the participating GPs all worked in the same health facility [7,10]. This may have produced a spill-over effect. There was a profound sampling variation in the included studies, for example: One study group chose to set up inclusion criteria for the GPs' participation in the study [9]. Another study group invited the participating GPs through ads in newspapers [8]. In one study, only 5% of the invited GPs participated in the study [13]. This variation in sampling makes comparison between the included studies difficult and meta-analysis irrelevant.

All studies were un-blinded to participating doctors, as allocation concealment was not possible. Some attempts of blinding were made in one study [17]. However, three study groups did anticipate the problem of contamination between intervention and control groups. One performed statistical analysis to investigate the size of the problem, which was seemingly insignificant [7]. Another study group argued against contamination due to lack of blinding in the study, based on recorded differences between control an intervention group, but without performing any statistical analysis of the size of the problem [8]. Lastly, one study group, as mentioned above, attempted blinding. However, the researchers themselves argue in the paper that the blinding was not extensive enough.

### Electronic feedback

Two trials complied with the definition of using electronic feedback [14,15]. Both trials obtained statistical significant effects on single measurements: Cholesterol measurement and blood pressure level, respectively. In addition, one of the trials demonstrated a statistical significant improvement of the compliance rate (Table 3).

## Discussion

### Main findings

This review demonstrates that it seems possible to improve the quality of diabetes care using feedback to the GPs. Significant positive changes are primarily seen on process, but also on outcome of Type 2 diabetes care. In many studies, large numbers of effect measures showed no significant change, but no study groups report deterioration in effect measures. In table 2 effect measures which showed significant positive changes are marked. All of the included studies leaned on national diabetes guidelines or developed diabetes guidelines for good control and care within the study.

### Implications of findings

The findings of this review indicate that feedback could be a valuable asset in quality improvement efforts. In the 2006 Cochrane Review referred to earlier in this paper, it was stated that "(...) the relative effectiveness of audit and feedback is likely to be greater when baseline adherence to recommended practice is low (...)" [3]. The results of this review support that statement to a certain extent: Among the endpoints reviewed, three endpoints showed significant improvement in more than one trial, namely numbers of foot examination made and the mean level of Hba1c and blood pressure. Foot examination is traditionally an endpoint with low guideline adherence. Mean level of Hba1c and blood pressure are influenced by life style changes and medication alterations, and so is very dependent on a strong commitment in the patient to alter their life style. Often, the strong dependency on life style changes can be unappealing to the doctors, because motivating the patient for life style changes is a difficult path to embark on [18].

### Strengths and weaknesses

The strength of this review is the specific aim limiting the searches to include only feedback trials on diabetes care in general practice. Ideally, this would optimise the homogeneity of the studies and strengthen the conclusions of the review.

One weakness was the risk of missing important trials. Papers written in other languages than English were excluded and it has been reported that such exclusion of trials in systematic reviews increases the likelihood of systematic errors and reduces precision [19].

It was not possible to perform meta-analysis on the findings due to heterogeneity of the studies included: The designs and methods of feedback were too inconsistent and the endpoints measured and the duration of the trials varied too much. The aim of all studies included however, was to improve diabetes care in general practice, according to the relevant guidelines. This common goal, paired with the delivering of feedback to GPs in all the studies included makes it relevant to draw conclusions based on this review.

### Delivering feedback

Clinicians have limited time to concentrate on quality improvement in daily practice which is why efforts to improve and sustain quality should be made as easily available and useable as possible [20]. Using computers is generally considered to be a positive thing saving time to the daily routines for GPs [21,22]. Considering this, it is striking that so few trials have tested electronic feedback on diabetes care in general practice.

### Evaluating feedback

An important point when evaluating research within the field of quality improvement is choosing the relevant effect measures [18]. The studies included in this review were not consistent in choosing effect measures.

Another issue is whether the changes detected in the effect measures are actually attributable to feedback. In this paper we included one obvious multifaceted intervention [12]. While multifaceted interventions certainly have their place in investigating quality improvement in diabetic care, it can be difficult to know exactly which efforts made the difference. We are aware that attributing the significant changes in the care of the PT2D in the included multifaceted intervention solely to the efforts of providing feedback to the GPs probably is an over-interpretation. However, the effort of providing GPs with continuous feedback was made, and therefore the intervention should be considered in this review.

In this study we have limited our search to randomized controlled trials (RCTs). This approach is debatable. One could argue that, while RCTs are often referred to as the gold standard of trial designs, it provides a one-shot effect, which often covers a short time frame of the patients medical history. It is evident, when researching within the field of chronic disease management, that long term effects of interventions are very relevant. However, follow-up over time and without specific interventions demonstrates huge improvements in diabetic care [23]. Thus, it is difficult in non-RCTs to be sure that the improvement in care is due to the specific intervention applied. Within the field of feedback on diabetes care to general practitioners, non-RCT long term effect research has revealed effects on process, outcome and the overall provision of diabetes care [24,25]. Even though these studies have not been included in this review due to design choices made, the results still relevantly support the conclusion that feedback on diabetes care to general practitioners can lead to better quality of care, and that this improvement of care could possibly be long term.

## Conclusion

We found that disease specific feedback to GPs has an effect on diabetes care in General Practice. Pointing out which effect measures that are mostly affected by the interventions proved difficult.

Even though feedback seems a promising tool for quality improvement, it was only possible to identify ten very heterogeneous studies that evaluated feedback to GPs on diabetes care in randomized, controlled trials. Only two trials evaluated electronic feedback in randomized trials and it was not possible to quantify the added effect of electronic feedback. Therefore, more research of this area is required.

## Abbreviations

GP: General practitioner; PT2D: People with type 2-diabetes; Boc: Based on own calculations on data extracted from paper; Duc: Data unavailable for further calculations; OR: Odds ratio; HR: Hazard ratio; Inh: inhibitor; Vac: vaccination; Exam: Examination.

## Competing interests

The authors declare that they have no competing interests.

## Authors' contributions

TLG co-designed the study, carried out searches and assessments, and drafted the manuscript. TL conceived of the study and participated in the writing process. JKK revised the manuscript and co-designed the study. PV co-designed the study, and was part of the assessments and the writing process. All authors have read and approved the final manuscript.

## Pre-publication history

The pre-publication history for this paper can be accessed here:


